# Genital Herpes Zoster: A Report of a Rare Case

**DOI:** 10.7759/cureus.72290

**Published:** 2024-10-24

**Authors:** Sofia Kalantzi, Chalent Alexakis, Maria Emmanouela Anagnostaki, Ismini Anagnostaki, Konstantinos Zacharis

**Affiliations:** 1 Department of Obstetrics and Gynecology, General Hospital of Lamia, Lamia, GRC; 2 Department of Dermatology, Papageorgiou General Hospital, Thessaloniki, GRC

**Keywords:** genital herpes zoster, genital lesions, varicella-zoster virus, vesicular rash, viral reactivation

## Abstract

Herpes zoster (HZ), caused by the reactivation of the varicella-zoster virus (VZV), typically presents as a unilateral vesicular rash in a dermatomal pattern. Its occurrence in the genital area is rare and often misdiagnosed. We report the case of a 51-year-old woman who presented with sudden vulvar pain and burning, without a history of immunodeficiency or sexually transmitted infections. Examination revealed unilateral vesicular lesions on the left labium majus, perianal area, medial thigh, and gluteal region along the second sacral (S2) and third sacral (S3) dermatomes. Polymerase chain reaction (PCR) confirmed VZV and ruled out herpes simplex virus (HSV). The patient was treated with brivudine, mupirocin, and analgesics, resulting in a complete resolution of symptoms. This case highlights the need to consider HZ in the differential diagnosis of genital lesions. Early recognition and treatment of atypical presentations can prevent complications and improve patient outcomes.

## Introduction

Varicella-zoster virus (VZV) was first isolated in 1954 by Weller and Coons from vesicular lesions of chickenpox and herpes zoster (HZ) [1]. In 1965, Hope-Simpson was the first to propose the hypothesis that VZV remains latent in the ganglia of sensory nerves after the initial infection. This hypothesis was later confirmed by histological studies, which found the virus in the nerve ganglia of the central nervous system [2]. Following reactivation and replication, the virus migrates through sensory nerve fibers to the dermatome associated with the affected ganglion. The entry of the virus into the keratinized cells of the dermatomes leads to the manifestation of the typical and characteristic rashes and lesions of recurrent diseases [3].

VZV is a herpesvirus, specifically human herpesvirus 3 (HHV-3), which frequently causes infections in humans. The primary infection with this virus results in the development of chickenpox [4]. During the primary infection, in a significant percentage of patients, the virus migrates to the ganglia, primarily the sensory nerve ganglia, where it integrates into the host cell DNA as segmental DNA [5]. In the ganglia, it remains in a latent state [6], but under the influence of nonspecific triggering factors (such as trauma, temperature changes, fever, infections, etc.), it can reactivate, leading to recurrent infections [7]. Reactivation can occur at any time, even decades after the primary infection [8].

While HZ typically presents as a unilateral vesicular rash along a dermatomal distribution, its occurrence in atypical locations, such as the genital area, is less commonly reported and can lead to significant clinical challenges [9]. Understanding the implications of such presentations is crucial, as they may mimic other dermatological or infectious conditions, leading to misdiagnosis and inappropriate management [10]. Through this report, we hope to contribute to the existing literature on HZ and encourage further discussion on its varied presentations, ultimately fostering a more comprehensive approach to patient care.

## Case presentation

**Figure 1:**
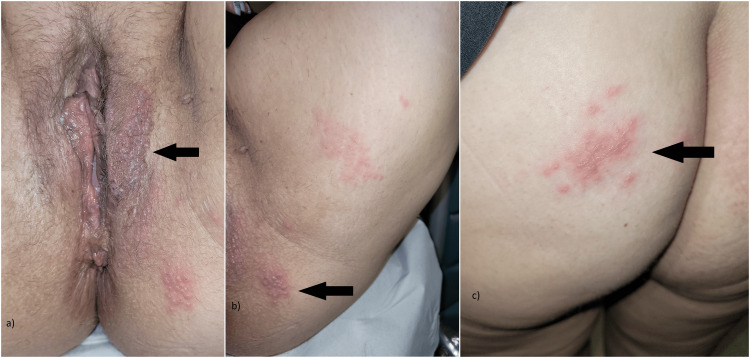
Vesicular lesions on the vulva, perianal, and thigh surfaces. The arrows point to the sites of the lesions: a) perivulvar, b) perianal, and c) gluteal

A 51-year-old woman presented to the gynecology emergency department with a one-day history of vulvar pain and burning sensation. She did not have an immune deficiency disorder and reported no history of herpes simplex virus (HSV) or prior sexually transmitted infections. No sexual risk behavior was reported. Gynecologic examination in the lithotomy position revealed unilateral vesicular lesions on an erythematous base on her left labium majus, the perianal area, the medial thigh surface, and the gluteal region (Figure 1). There was no associated lymphadenopathy.

The eruption extended along the second sacral (S2) and third sacral (S3) dermatomes, and no associated localized lymphadenopathy was detected. The physical examination of the patient did not reveal any abnormalities. Temperature and vital signs were normal. A pelvic abdominal ultrasound was ordered, which was also normal. Complete blood count, urinalysis, and liver function tests were all within the normal range. Several serological markers were ordered to exclude conditions from the differential diagnosis. Anti-human immunodeficiency virus (HIV) antibodies, hepatitis B surface antigen, anti-hepatitis C virus antibodies, and the Venereal Disease Research Laboratory (VDRL) test were all negative. Molecular testing of the blister fluid with polymerase chain reaction (PCR) analysis was negative for HSV types 1 and 2 and positive for VZV. The bacterial culture was also negative. The patient had a history of chickenpox in childhood, was not vaccinated for it, and had not experienced any subsequent episodes of varicella, making this her first reported episode since childhood. Hence, the patient was diagnosed with genital HZ, and she was immediately treated with brivudine 125 mg once daily for seven days along with local mupirocin application and analgesic drugs. After one week, the follow-up assessment showed significant improvement in symptoms. Upon examination, the lesions were in various stages of healing, and no new ulcerative or vesicular lesions were observed. The patient was advised to be vaccinated for varicella and was referred to an infectious disease specialist for further information.

## Discussion

VZV, a neurotropic virus, travels centripetally along sensory nerve endings from infected cutaneous and mucosal lesions to dorsal root and cranial nerve ganglia after the resolution of primary varicella infection in childhood and remains in the latent phase [1]. HZ is an infection that occurs when VZV is reactivated from its latent state in a posterior dorsal root ganglion. Due to the rarity of VZV in the genital area, most initial diagnoses were HSV infections since they have similar symptomatology and morphology, resulting in a higher reported incidence of HZ than what is typically documented in the literature [11]. The clinical symptoms of HZ include a painful and erythematous papular rash that occurs in a dermatomal distribution [12]. Lesions are typically unilateral, as observed in our case.

The occurrence of HZ in the female genital region is a rare complication of VZV infection. Epidemiological documentation of specific cases of HZ in the genital area is limited, primarily due to the rarity of this localization. Because of the infrequency of these cases, data regarding the exact epidemiology of HZ in the genital area is scarce and typically recorded only in isolated studies and case reports [13].

Varicella can result in up to a 90% infection rate in non-immune individuals, with the incidence increasing in immunocompromised patients [14]. Distinguishing HZ from other vesicular eruptions of the vulva can be challenging, as the symptoms may overlap. A key diagnostic approach is isolating the virus from vesicular fluid by culture, as performed in our case [12]. Cultivation of the virus from lesions remains the best and most reliable method, with positivity rates of 80-99% during primary infection and typically done within approximately 2-7 days [15]. However, in cases of recurrent genital herpes, the likelihood of a positive culture is significantly lower, not exceeding 40% [16]. Thus, a negative culture does not rule out genital herpes, and it may be necessary to culture multiple lesions for a definitive diagnosis. The PCR method for detecting viral DNA in vesicular fluid has proven to be highly sensitive, reliable, and quick, and it may eventually replace culture as the preferred diagnostic method for genital herpes [17].

Differentiating HZ in the genital area from other conditions is essential for effective management. Common differential diagnoses include herpes simplex virus (HSV) infection, which presents with painful vesicular lesions and can be confirmed through PCR testing; contact dermatitis, characterized by pruritic lesions and identifiable allergens via patch testing; scabies, which shows intensely itchy papules confirmed by microscopic examination; and folliculitis, typically presenting with pustules around hair follicles and diagnosed through bacterial cultures [18].

Treatment should commence as soon as possible to prevent bacterial infection of rashes and to provide faster relief of pain. The initiation of antiviral therapy within 72 hours of rash onset has been shown to significantly reduce the severity and duration of symptoms, minimizing the risk of complications such as secondary bacterial infections. The most common complication is postherpetic neuralgia (PHN), which can persist for several months and severely impact the patient's quality of life [14]. Early intervention not only helps alleviate acute pain but also reduces the likelihood of developing PHN, thus improving long-term outcomes for patients. Vaccination remains the primary preventive measure against varicella and, consequently, HZ, as it effectively reduces the incidence and severity of both diseases [18].

## Conclusions

In summary, this case report highlights the significant impact of HZ in the genital region, emphasizing the need for increased awareness and understanding of this condition among healthcare providers. The presentation of HZ in this area can cause considerable discomfort and psychological distress for patients, often complicating diagnosis and management. Our findings suggest that early recognition and appropriate antiviral treatment are crucial for mitigating symptoms and preventing complications. Furthermore, this case underscores the importance of considering HZ in the differential diagnosis of genital lesions, even in patients without a history of varicella infection. Continued research and education are essential to enhance clinical outcomes and improve the quality of life for those affected by this condition.

## References

[1] Blattner R (1954). Varicella and herpes zoster. J Pediatr.

[2] Reichelt M, Zerboni L, Arvin AM (2008). Mechanisms of varicella-zoster virus neuropathogenesis in human dorsal root ganglia. J Virol.

[3] Hunt JR (1968). On herpetic inflammations of the geniculate ganglion. A new syndrome and its complications. Arch Neurol.

[4] Gnann JW Jr, Whitley RJ (2002). Clinical practice. Herpes zoster. N Engl J Med.

[5] Gilden DH, Vafai A, Shtram Y, Becker Y, Devlin M, Wellish M (1983). Varicella-zoster virus DNA in human sensory ganglia. Nature.

[6] Eshleman E, Shahzad A, Cohrs RJ (2011). Varicella zoster virus latency. Future Virol.

[7] Hope-Simpson RE (1965). The nature of herpes zoster: a long-term study and a new hypothesis. Proc R Soc Med.

[8] Marinelli I, van Lier A, de Melker H, Pugliese A, van Boven M (2017). Estimation of age-specific rates of reactivation and immune boosting of the varicella zoster virus. Epidemics.

[9] Magdaleno-Tapial J, Hernández-Bel P, Ortiz-Salvador JM (2022). Genital herpes zoster: a rare location that can mimic genital herpes. Sex Transm Dis.

[10] Chiriac A, Chiriac AE, Naznean A, Moldovan C, Podoleanu C, Stolnicu S (2019). Sacral (S1) herpes zoster. J Pain Res.

[11] Harbecke R, Oxman MN, Selke S, Ashbaugh ME, Lan KF, Koelle DM, Wald A (2024). Prior herpes simplex virus infection and the risk of herpes zoster. J Infect Dis.

[12] Kennedy PG, Gershon AA (2018). Clinical features of varicella-zoster virus infection. Viruses.

[13] Magdaleno-Tapial J, Hernández-Bel P, Ortiz-Salvador JM, López-Martí C, Martínez-Doménech Á, García-Legaz-Martínez M, Pérez-Ferriols A (2022). Genital herpes zoster: a rare location that can mimic genital herpes. Sex Transm Dis.

[14] Cook B, Shadowen C, Clark L, Hoover A, Lee S, Bender W (2024). Genital VZV in a third trimester pregnancy and the critical role of interdisciplinary planning. Case Rep Infect Dis.

[15] Corey L, Holmes KK (1983). The current status of herpes simplex virus infections in the newborn. Am J Dis Child.

[16] Prober CG, Sullender WM, Yasukawa LL, Au DS, Yeager AS, Arvin AM (1987). Low risk of herpes simplex virus infections in neonates exposed to the virus at the time of vaginal delivery to mothers with recurrent genital herpes simplex virus infections. N Engl J Med.

[17] Nath P, Kabir MA, Doust SK, Ray A (2021). Diagnosis of herpes simplex virus: laboratory and point-of-care techniques. Infect Dis Rep.

[18] Saguil A, Kane S, Mercado M, Lauters R (2017). Herpes zoster and postherpetic neuralgia: prevention and management. Am Fam Physician.

